# Sir2 suppresses transcription-mediated displacement of Mcm2-7 replicative helicases at the ribosomal DNA repeats

**DOI:** 10.1371/journal.pgen.1008138

**Published:** 2019-05-13

**Authors:** Eric J. Foss, Tonibelle Gatbonton-Schwager, Adam H. Thiesen, Erin Taylor, Rafael Soriano, Uyen Lao, David M. MacAlpine, Antonio Bedalov

**Affiliations:** 1 Clinical Research Division, Fred Hutchinson Cancer Research Center, Seattle, WA, United States of America; 2 Department of Pharmacology and Cancer Biology, Duke University, Durham, NC, United States of America; 3 Department of Medicine, University of Washington, Seattle, WA, United States of America; 4 Department of Biochemistry, University of Washington, Seattle, WA, United States of America; University of California San Francisco, UNITED STATES

## Abstract

Repetitive DNA sequences within eukaryotic heterochromatin are poorly transcribed and replicate late in S-phase. In *Saccharomyces cerevisiae*, the histone deacetylase Sir2 is required for both transcriptional silencing and late replication at the repetitive ribosomal DNA arrays (rDNA). Despite the widespread association between transcription and replication timing, it remains unclear how transcription might impinge on replication, or *vice versa*. Here we show that, when silencing of an RNA polymerase II (RNA Pol II)-transcribed non-coding RNA at the rDNA is disrupted by *SIR2* deletion, RNA polymerase pushes and thereby relocalizes replicative Mcm2-7 helicases away from their loading sites to an adjacent region with low nucleosome occupancy, and this relocalization is associated with increased rDNA origin efficiency. Our results suggest a model in which two of the major defining features of heterochromatin, transcriptional silencing and late replication, are mechanistically linked through suppression of polymerase-mediated displacement of replication initiation complexes.

## Author summary

Eukaryotic genomes typically contain large regions of repetitive DNA, referred to as heterochromatin, that are both transcriptionally silent and late replicating. We provide a possible explanation for the association between transcriptional silencing and late replication. Budding yeast contains a histone deacetylase called *SIR2* that was originally identified as a transcriptional repressor, but was later also found to ensure late replication of repetitive ribosomal DNA (rDNA) sequences. We show that the transcription that occurs in the absence of *SIR2* directly displaces the helicase required for replication initiation at the rDNA. This work represents an important advance in understanding the interplay between transcription and replication at repetitive sequences by directly linking transcription with replication machinery in heterochromatin.

## Introduction

Approximately half of the human genome consists of repetitive DNA sequences organized as heterochromatin. These regions are largely devoid of genes and are characterized by both low levels of transcription and late DNA replication [1–4]. The association between low levels of transcription and late replication is well established and extends to regions of the genome that are transcriptionally active only during specific stages of development. In stages of development when genes within these regions are transcribed, they replicate early, and when these genes are no longer expressed, their replication is delayed [5, 6]. In contrast, so-called "housekeeping" genes, which are constitutively transcribed, replicate early during all stages of development. Replication timing has important evolutionary implications for genome stability, with late replicating regions being more prone to mutation and rearrangement [7, 8]. Despite the prevalence and evolutionary significance of the association between transcription and replication timing, its mechanistic underpinnings remain elusive. It has been proposed that differences in histone modifications or nuclear localization between heavily transcribed and silenced genome regions may affect their replication timing, but it has proved difficult to establish mechanistic foundation for these associations [9, 10].

**Fig 1:**
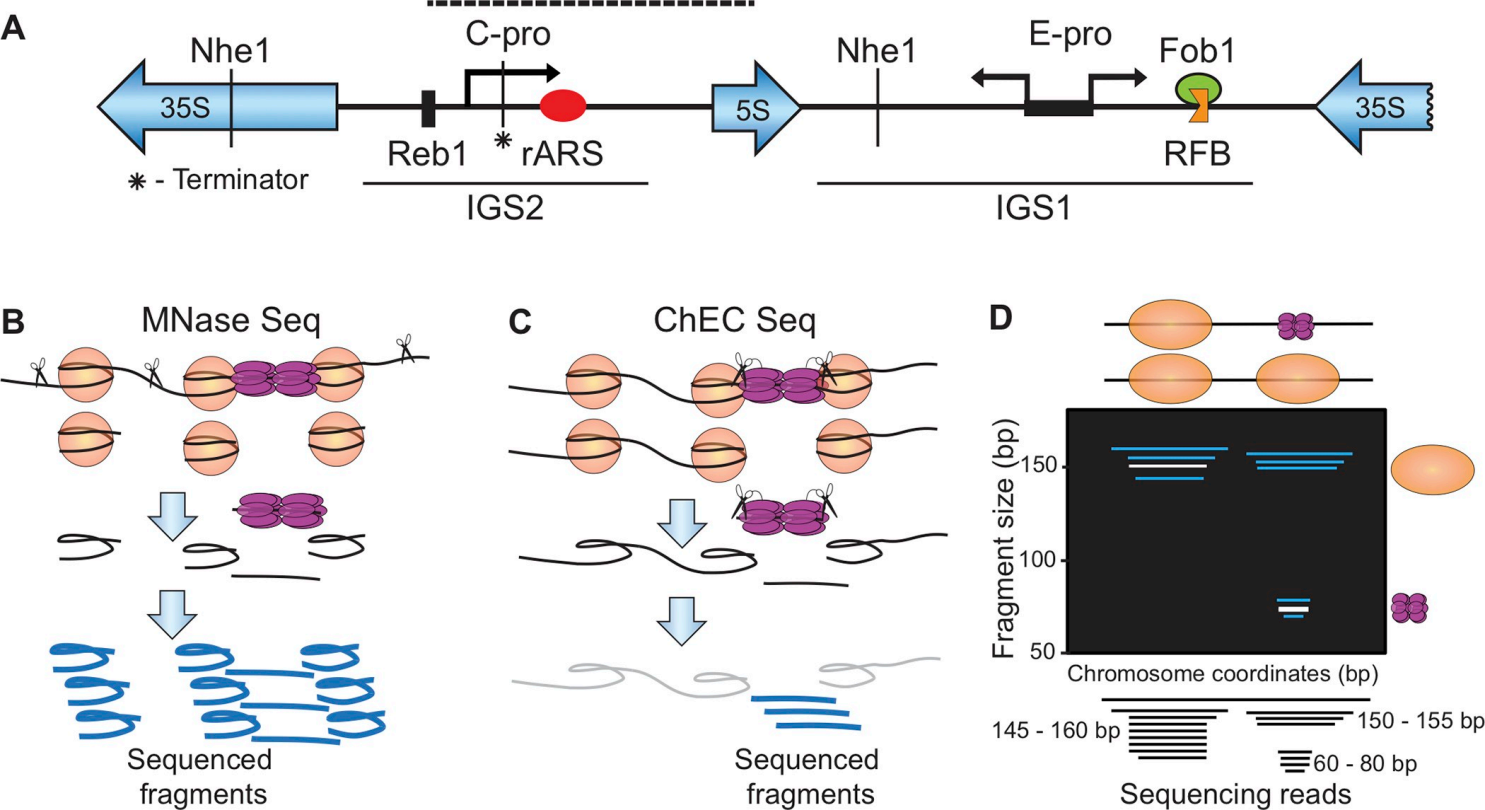
Ribosomal rDNA locus and sequencing-based approaches to chromatin analysis. A. Landmarks of the *S*. *cerevisiae* rDNA repeat. Each 9.1 kb repeat contains rRNA genes for the Pol I-transcribed 35S precursor RNA and the Pol III-transcribed 5S RNA, separated by two intergenic spacer regions, *IGS1* and *IGS2*, both of which are subject to Sir2-mediated silencing. *IGS1* harbors the bidirectional Pol II promoter e-pro and the replication fork barrier (RFB), a binding site for Fob1, which enforces a unidirectional block to DNA polymerase to reduce DNA polymerase and RNA Pol I collision. *IGS2* harbors c-pro, a Sir2-repressed Pol-II promoter, and rARS located 200 bp downstream from the c-pro initiation site. Additional markings include the binding site for the transcription factor Reb1 immediately upstream of c-pro, the dashed line marking the 1.25 kb fragment containing rARS analyzed in Figs 2–4, the restriction enzyme Nhe1 sites, used in 2D gels in Fig 5, and the insertion site for the terminator, used in Fig 6. B. MNase-seq enables identification of footprints of nucleosomes and other DNA binding proteins by virtue of exogenously added MNase degrading DNA that is not protected by binding of such proteins, followed by library preparation and sequencing. C. ChEC-seq involves tagging a DNA-binding protein of interest (Mcm2 shown) followed by activation of the nuclease with calcium and library preparation and sequencing. Only the MNase-cleaved fragments of interest are short enough to be PCR amplified during library preparation. Large circle presents a nucleosome, purple doublet presents Mcm2-7 and the scissors represent MNase. D. Presentation of sequencing data from MNase-seq and ChEC-seq experiments. Each mapped read is graphed according to its genomic location, on the X-axis, and its size, on the Y axis. When two particles of different sizes (e.g. a nucleosome and a replication initiation factor) occupy the same genomic region in different populations of cells, they can be distinguished by differently sized footprints that map to the same location.

In *S*. *cerevisiae*, repetitive regions within the rDNA locus and at telomeres are subject to regional, gene-independent transcriptional silencing and are considered simple models of the heterochromatin found in higher eukaryotes (reviewed in [11, 12]). In a further parallel to metazoan heterochromatin, both of these repetitive regions replicate late in the cell cycle [13–15]. The NAD-dependent histone deacetylase Sir2 is required for heterochromatin formation at both sites, but with different partners at the two locations, forming RENT (REgulator of Nucleolar silencing and Telophase exit) and SIR (Silent Information Regulator) complexes at the rDNA and telomeres, respectively [16, 17]. The rDNA locus in yeast consists of 150 tandemly arranged 9.1 kb repeats that occupy half of chromosome XII and comprise 10% of total genomic DNA. Each repeat contains ribosomal rRNA genes for the Pol I-transcribed 35S precursor RNA and for the Pol III-transcribed 5S RNA, separated by two intergenic spacer regions (*IGS1* and *IGS2*) (Fig 1A). The spacer regions are subject to Sir2-dependent silencing and harbor two Pol II promoters, c-pro and e-pro, that drive transcription of non-coding RNAs. Two hundred base pairs downstream from c-pro, within *IGS2*, is the ribosomal Autonomously Replicating Sequence ("rARS"). The rARS serves as a replication origin, whose activation is also suppressed by Sir2. Thus, *SIR2* imposes both transcriptional silencing and replication origin repression at the rDNA. This feature, together with the homogeneous nature of the repeats, make the yeast rDNA a highly suitable model for examining the relationship between transcriptional silencing and replication in heterochromatin.

The rARS serves as a binding site for the origin recognition complex (ORC), which assists with loading the Mcm2-7 complex (Mcm2-7) at rDNA origins. Because the rARS is located immediately downstream of the initiation site for a Sir2-regulated ncRNA, we reasoned that the transcription machinery may alter the deposition of replication initiation factors at the rDNA origins, which is referred to as origin licensing. Specifically, we hypothesized that RNA Pol II passage through the rDNA origin might either promote loading of Mcm2-7 replicative helicases or, in light of a previous reports [18, 19], induce their sliding along DNA and re-localization away from their loading site. Further supporting the possibility that Sir2 affects origin licensing is our observation that excessive origin activation in *sir2* mutants can be suppressed by a point mutation in the origin recognition complex (Orc)-binding site within the rARS [13, 20]. Here we tested the idea that transcription alters deposition of the pre-replicative complex (pre-RC) at rDNA origins by using sequencing-based methods to obtain and compare high-resolution footprints of nucleosomes and replication initiation factors at the rDNA origins in wild type (WT) and *sir2* mutant cells. This analysis revealed that disruption of transcriptional silencing upon *SIR2* deletion leads to RNA Pol II-mediated displacement of pre-RCs away from their loading site at the rDNA origins, which effectively repositions them from an area with high nucleosome occupancy to one with low. While our studies do not prove causality in the association between pre-RC repositioning and advanced replication timing, given that the overall abundance of pre-RCs at the rDNA is reduced in *sir2* cells, we propose a model in which repositioning of pre-RCs to regions with low nucleosome occupancy in *sir2* cells facilitates their subsequent activation.

## Results

### High resolution pre-RC footprints at the rDNA

To determine whether the absence of *SIR2*-mediated transcriptional repression alters chromatin architecture at the rARS, we sought to profile the chromatin organization of this locus at nucleotide resolution both before and after pre-RC assembly at the rARS. Chromatin perturbations dependent on pre-RC assembly should be (1) G1-specific; (2) *CDC6*- and *ORC1*-dependent; and thus reflective of binding of the Mcm2-7 helicase complex.

We first used a micrococcal nuclease (MNase)-based high-resolution epigenome profiling technique called MNase-Seq (Fig 1B) to assess the chromatin architecture in a factor-agnostic manner. This technique reveals DNA footprints protected by the histone octamer (nucleosomes) as well as smaller footprints protected by DNA-binding factors such as transcription and replication factors [21–23]. Briefly, total chromatin is digested with MNase and the resulting fragments are subjected to paired-end sequencing using a protocol that retains DNA fragments as small as 50 base pairs. The length of each fragment is plotted as a function of its chromosomal position; thus, the size of the fragment is indicative of the protein 'footprint' (Fig 1D). For example, DNA fragments protected by a histone octamer will be approximately ~150 bp and smaller fragments (50–100 bp) represent footprints of site-specific DNA-binding factors (Fig 1D).

**Fig 2:**
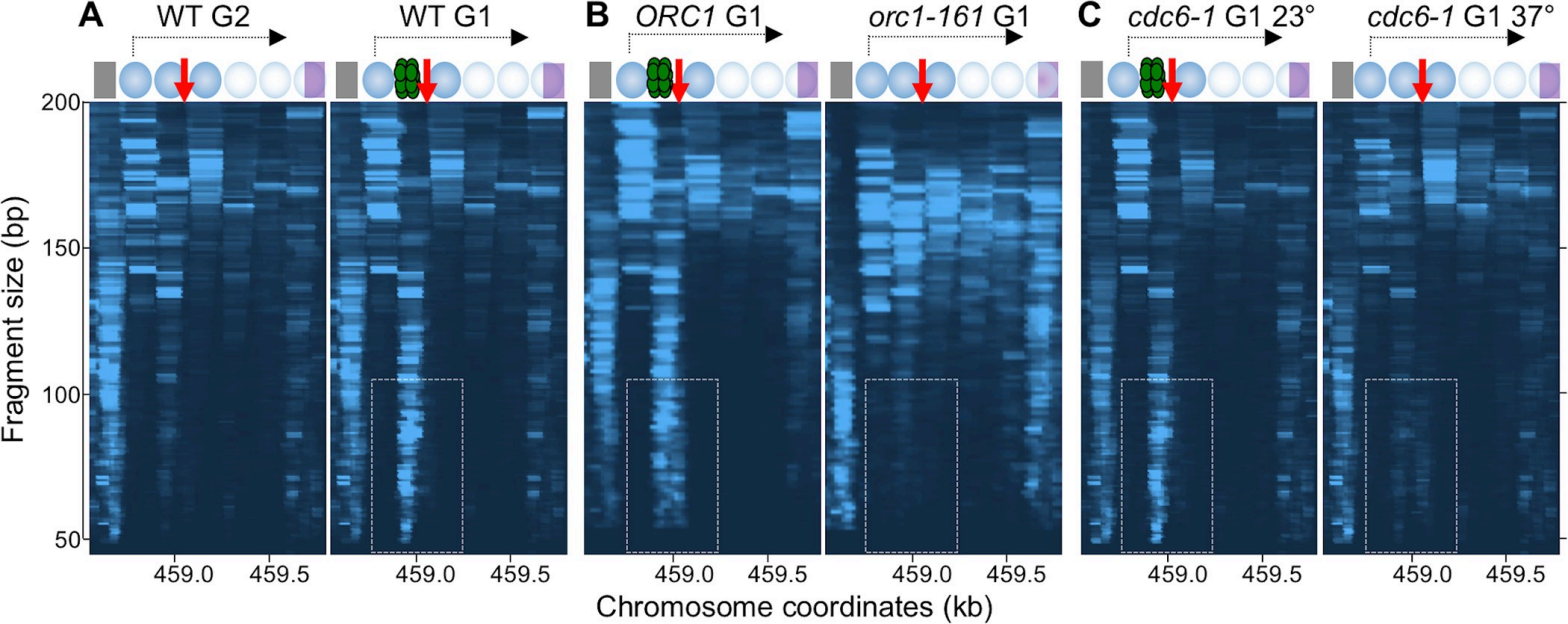
Pre-RC binding at rDNA origin is G1-specific and *ORC1*- and *CDC6*-dependent as determined by MNase-seq. Diagrams at the top of each panel denote a 1.25 kb region of the rDNA that includes the rDNA origin marked by a dashed line in Fig 1A. The gray rectangle on the left represents the Reb1 binding site (S1 Fig), blue circles represent nucleosomes with transparency level inversely related to their occupancy, the red vertical arrow shows the location of rARS, the green doublet represents the Mcm2-7 double hexamer, the purple rectangle represents the 5S RNA gene, and the black horizontal arrow represents the c-pro transcript. Genomic coordinates according to the sacCer3 genomic sequence are shown on the x axes, fragment length on the y axes and relative read depth is indicated by color intensity. Dashed rectangles point to the area that corresponds to the footprint of the Pre-RC complex. A. Chromatin was isolated from cultures of a WT strain (16535) arrested in G2 with nocodazole or in G1 with alpha factor, treated with MNase to digest DNA not protected by bound proteins and subjected to paired end sequencing. Plot shows genomic coordinate on the X axis, fragment size on the Y axis, and relative read depth indicated by color intensity. B. The analysis of previously published *orc1-161* MNase-seq data set [23]. *orc1-161* cells were arrested in G1 at the restrictive temperature and compared to *ORC1* cells. C. *cdc6-1* (16738) cells were arrested in G1 at permissive (23°C; left) or restrictive (37°C; right) temperature and processed as in A.

To gain insight into chromatin organization at the rDNA origins before the pre-RC is formed, we carried out MNase-seq on chromatin from cells arrested in the G2 phase of the cell cycle and plotted the reads that map to the 1.25 kb of rDNA sequence marked by a dashed line in Fig 1A that includes the replication origin. From left to right, we first observed a 50–100 bp footprint corresponding to the transcription factor Reb1 (gray rectangle in Fig 2A and S1 Fig), followed by the footprints of six nucleosomes in the 150–200 size range; the first three are particularly well positioned, and the last maps to the 5S RNA sequence. The first nucleosome, which coincides with the initiation site of the Sir2-repressed c-pro transcript exhibited highest occupancy, followed by the third one, whereas the occupancy of the last three nucleosomes averaged only 10% of the first one. Overall, these results underscore the highly organized nature of chromatin at the rDNA origins of replication, which is remarkably uniform considering that the footprints are a composite of many cells with a multitude of rDNA repeats.

We next used the same assay to analyze chromatin from cells arrested in G1, when the pre-RC forms. We observed a striking 50–100 bp footprint adjacent to the rARS (dashed rectangle in Fig 2A, right panel) suggestive of the pre-RC. To determine whether the formation of this G1-specific footprint is ORC-dependent, we analyzed a previously published MNase-seq data set for the *orc1-161* temperature-sensitive mutant [23]. We observed a loss of the footprint in the *orc1-161* mutant at the restrictive temperature (Fig 2B), as expected if the footprint constitutes the pre-RC. Because loading of the pre-RC requires Cdc6 [24], we used the temperature-sensitive allele *cdc6-1* [25] to determine whether the G1-specific signal we saw was Cdc6-dependent. Consistent with it reflecting pre-RC binding, the footprint was present in G1 at the permissive temperature of 23° but not at the restrictive temperature of 37° (dashed rectangle in Fig 2C). The G1-specificity and ORC- and Cdc6-dependence of this footprint, coupled with its localization adjacent to the rARS demonstrate that this footprint reflects pre-RC formation at the rDNA origins, and that MNase-seq constitutes an accurate and robust assay for examining chromatin changes associated with pre-RC formation at this location.

### Licensing of rDNA origins requires nucleosome eviction

**Fig 3:**
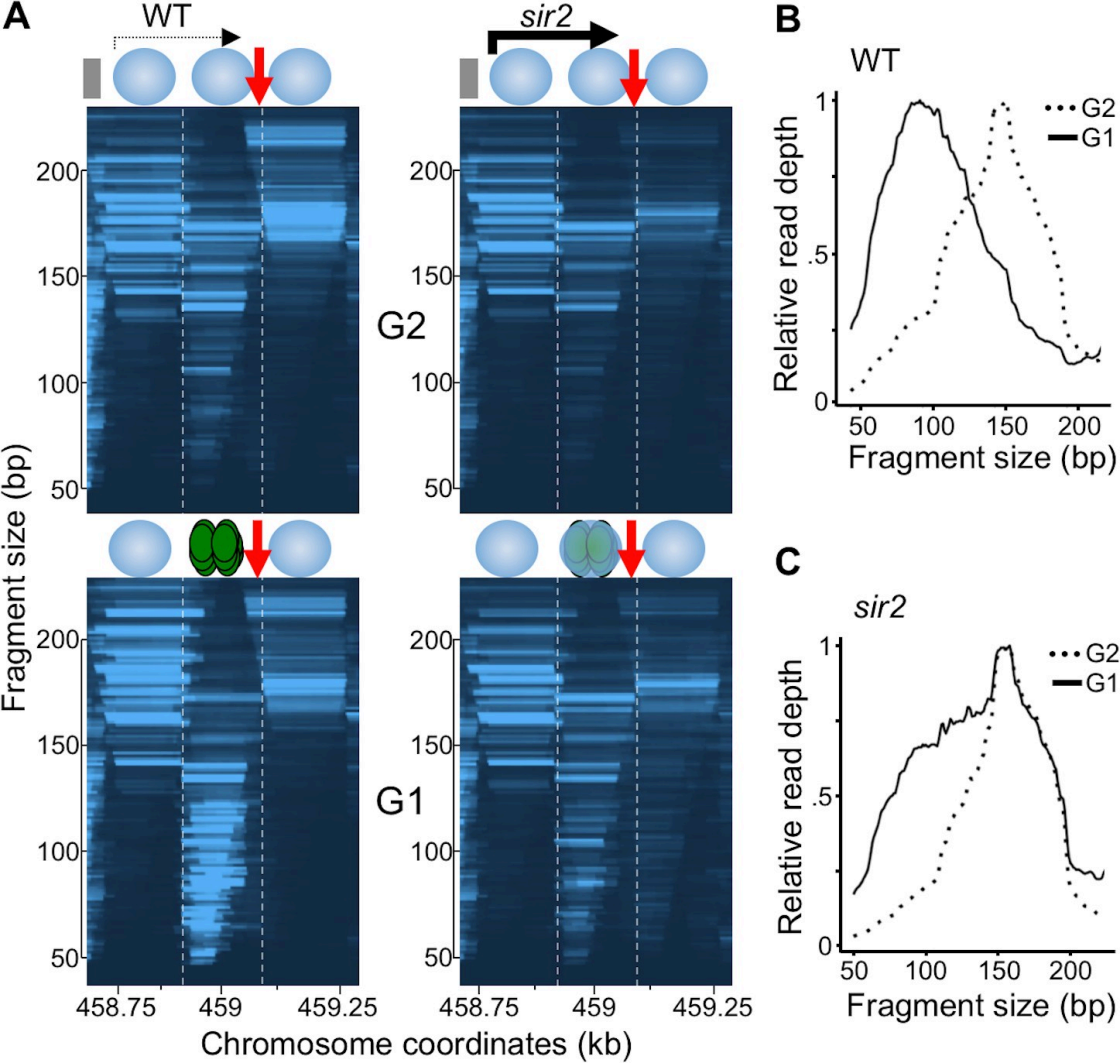
Pre-RC formation is associated with the eviction of the nucleosome at the rDNA origin. A. MNase-seq data from WT (16535) or *sir2* (16668) cells arrested in G2 or G1 are shown zoomed in to allow assessment of nucleosome positioning and occupancy. Vertical lines are positioned at the border of the middle nucleosome to facilitate comparison of nucleosome placement. A nucleosome between the vertical lines competes with the pre-RC for occupancy. Positioning of this nucleosome and the two flanking nucleosomes does not change between G1 and G2. Relative read depth in the genomic region between the two vertical lines in 3A on the Y axis, is plotted according the fragment size (X-axis) in WT (B.) and *sir2* mutant (C.). The large fragment sizes, that peak at approximately 160 bp correspond to nucleosome, whereas smaller fragments correspond to the pre-RC. In WT cells the nucleosome size fragments are depleted in G1 compared to G2, whereas they persist in *sir2* mutant.

Our MNase-seq results reveal binding dynamics of both the pre-RC and nucleosomes averaged across many cells and many rDNA repeats in each cell. Replication origins normally occur in "nucleosome-free regions" (NFRs) but, to our surprise, this was not the case at the rARS: Instead, our data showed a nucleosome precisely at the site of pre-RC formation (marked by dashed lines in Fig 3A) [23, 26, 27]. Furthermore, the pre-RC and this nucleosome compete for binding: In G1 the pre-RC signal is prominent while the nucleosome signal is faint, whereas the converse is true in G2 (Fig 3A). The coupling of the eviction of the nucleosomes with loading of the pre-RC in G1 is evident by changes in fragment size distribution at the sites of pre-RC loading (Fig 3B), with the size shifting from that corresponding to nucleosomes in G2, to the smaller size corresponding to pre-RC in G1. Finally, the two nucleosomes that flank the rARS do not appear to shift away from the origin to make space for binding of the pre-RC in G1. These results demonstrate that binding of the pre-RC and binding of the nucleosome at the rARS are mutually exclusive events, and that licensing of rDNA origins requires, or causes, eviction of this nucleosome.

### *SIR2* deletion displaces Mcm2-7 from their loading sites and decreases their overall abundance at the rDNA

**Fig 4:**
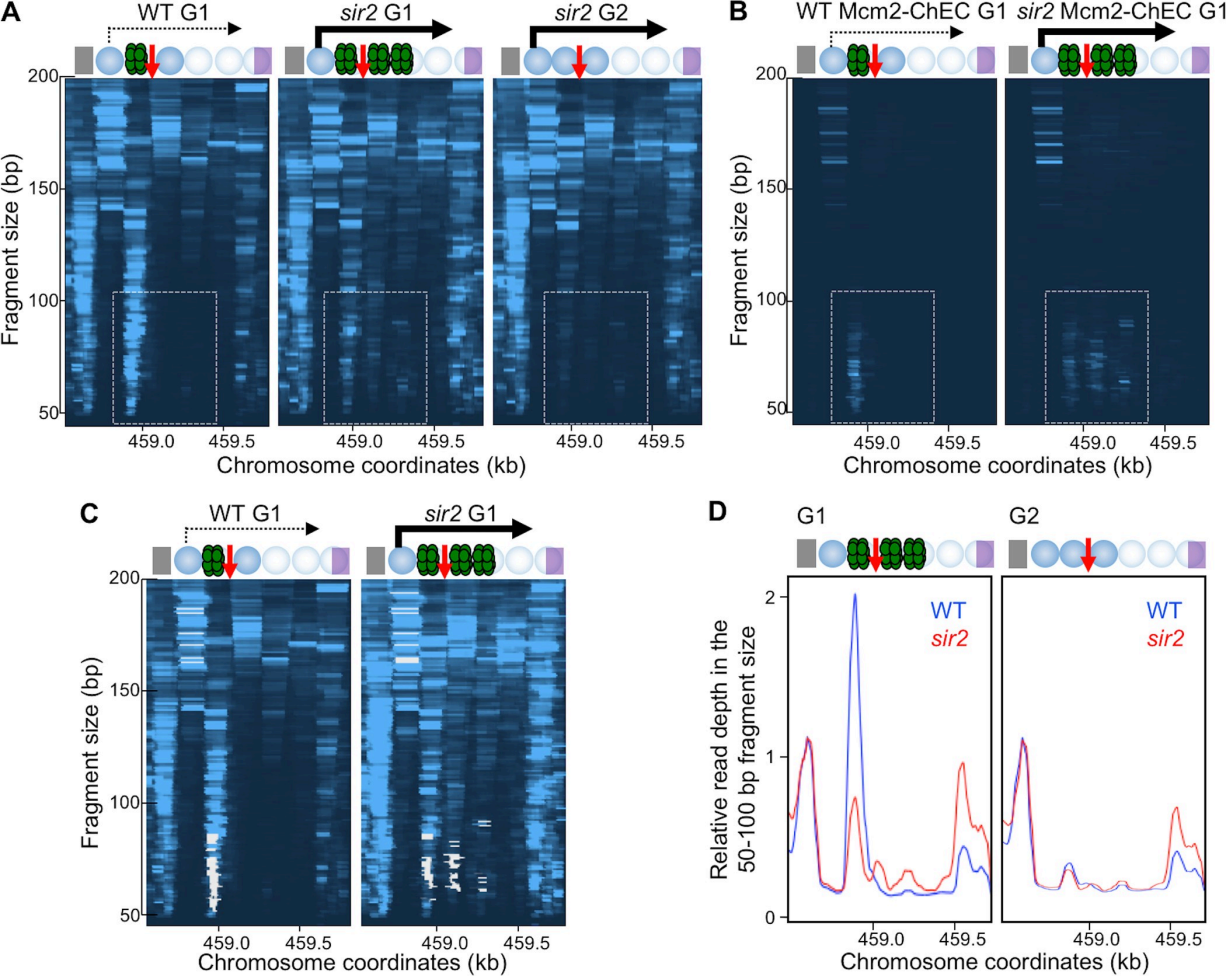
*SIR2* deletion repositions Mcm2-7. A. MNase-seq analysis. WT (16535) and *sir2* (16668) cells were arrested in G1 or G2 and processed as in Fig 2. B. Mcm2-MNase ChEC-seq analysis. G1-arrested, WT (WT) (16747) and *sir2* (16769) mutant cells, whose endogenous copy of MCM2 was tagged with MNase, were permeabilized with digitonin, treated with calcium for 30 seconds to activate MNase, and then processed and plotted as in Fig 2. Dashed rectangles point to the area corresponding to the Mcm2-7 footprint. C. Composite of images in 4A and 4B, with 4B shown in white. D. Quantitation of read depths from fragments 50–100 base pairs long from WT (blue) (16535) and *sir2* (red) (16668) cells arrested in G1 (left) or G2 (right) and otherwise treated as in Fig 2. Read depths were normalized to the read depth at the Reb1 binding site.

We next used MNase-seq to determine whether *SIR2* deletion increases pre-RC binding or changes the distribution of pre-RC and nucleosome occupancy at the rDNA. When we compared pre-RC footprints in WT and *sir2* cells arrested in G1, rather than seeing *increased* pre-RC binding and *decreased* nucleosome occupancy at the rARS in *sir2* compared to WT, as might be expected from the observation that *SIR2* deletion promotes rDNA replication, we instead saw the opposite: *SIR2* deletion led to *decreased* pre-RC binding (dashed rectangle in Fig 4A) and *increased* nucleosome occupancy (Fig 3A and 3C). Notably, however, the area to the right of the rARS, which is devoid of pre-RC binding in the WT, showed obvious binding in the 50–100 bp DNA fragment size range in the *sir2* mutant (Fig 4A). These 50–100 bp footprints in *sir2* cells (dashed rectangle in Fig 4A), including both those on the left and on the right of rARS, were G1-specific, consistent with pre-RC binding pattern we have previously observed in WT cells. These results suggest that the pre-RC might be displaced rightward in *sir2* mutant, perhaps thereby allowing a nucleosome to occupy the vacated space at the rARS.

The Mcm2-7 helicase is a major component of the pre-RC. To determine whether the pre-RC footprint we observed by MNase-seq contains Mcm2-7, and to confirm its redistribution in the *sir2* mutant, we tagged the endogenous copy of Mcm2 at its C-terminus with MNase and carried out the "Chromatin Endogenous Cleavage" assay (ChEC) (Fig 1C) [28, 29]. Mcm2 function was not altered by the presence of the tag as judged by comparable growth of WT and Mcm2-tagged strain (S2 Fig). In ChEC, a short burst of calcium-activated MNase activity induces *in situ* cleavage of DNA adjacent to the fusion protein. We prepared sequencing libraries from the cleaved DNA, again using a protocol that retains small DNA fragments and presented the sequencing data in two dimensions as for the MNase-seq results (Fig 4B). The footprint we obtained coincided with the G1-specific, *CDC6*- and *ORC1*- dependent footprint observed by MNase-seq (Fig 4B and 4C). Furthermore, we observed that the Mcm2-7 footprints in *sir2* cells can be also found right of the rARS, whereas they are confined left of the rARS in the WT cells (Fig 4B). The Mcm2-7 footprints to the right of the rARS in the *sir2* mutant were not suppressed by deletion of *FOB1* (S4 Fig), indicating that they do not originate from extrachromosomal rDNA circles that accumulate in a *sir2* mutant in a *FOB1*-dependent manner [30]. These results suggested that the bound Mcm2-7, assuming it is loaded by ORC at the same site in both WT and *sir2*, is pushed rightward in the *sir2* mutant. This movement effectively repositions Mcm2-7 from an area of high nucleosome occupancy to one with low.

We next quantified abundance of Mcm2-7 in WT and *sir2* mutant using both our MNase-seq and ChEC-seq datasets. In the MNase-seq dataset we integrated read depths in the 50–100 base fragment size range in the region that encompasses both displaced and non-displaced Mcm2-7, and normalized it to read depths corresponding the footprint of the transcription factor Reb1 (gray rectangle in Fig 4A). The *sir2* mutant exhibited a 37% reduction in Mcm2-7 binding relative to WT (Fig 4D). To calculate Mcm2-7 abundance using ChEC-seq, we compared the fraction of footprints that map to rDNA, among all Mcm2-7 footprints, in WT and *sir2* cells. We observed a 47% reduction in Mcm2-7 binding in this region in *sir2* vs WT in our Mcm2-MNase ChEC dataset, which is similar to what we have observed using MNase-seq dataset. We conclude that the overall abundance Mcm2-7 complexes at the rDNA is markedly reduced in *sir2* mutants.

A combination of the findings that Mcm2-7 complexes are repositioned in a *sir2* mutant to an area with low nucleosome occupancy and that overall Mcm2-7 binding at the rDNA is lower in a *sir2* mutant compared to WT suggests that accelerated replication of the rDNA in the absence of *SIR2* is a consequence of pre-RC repositioning rather than increased origin licensing.

### 2D gel analysis of replication initiation in *sir2* mutant

**Fig 5:**
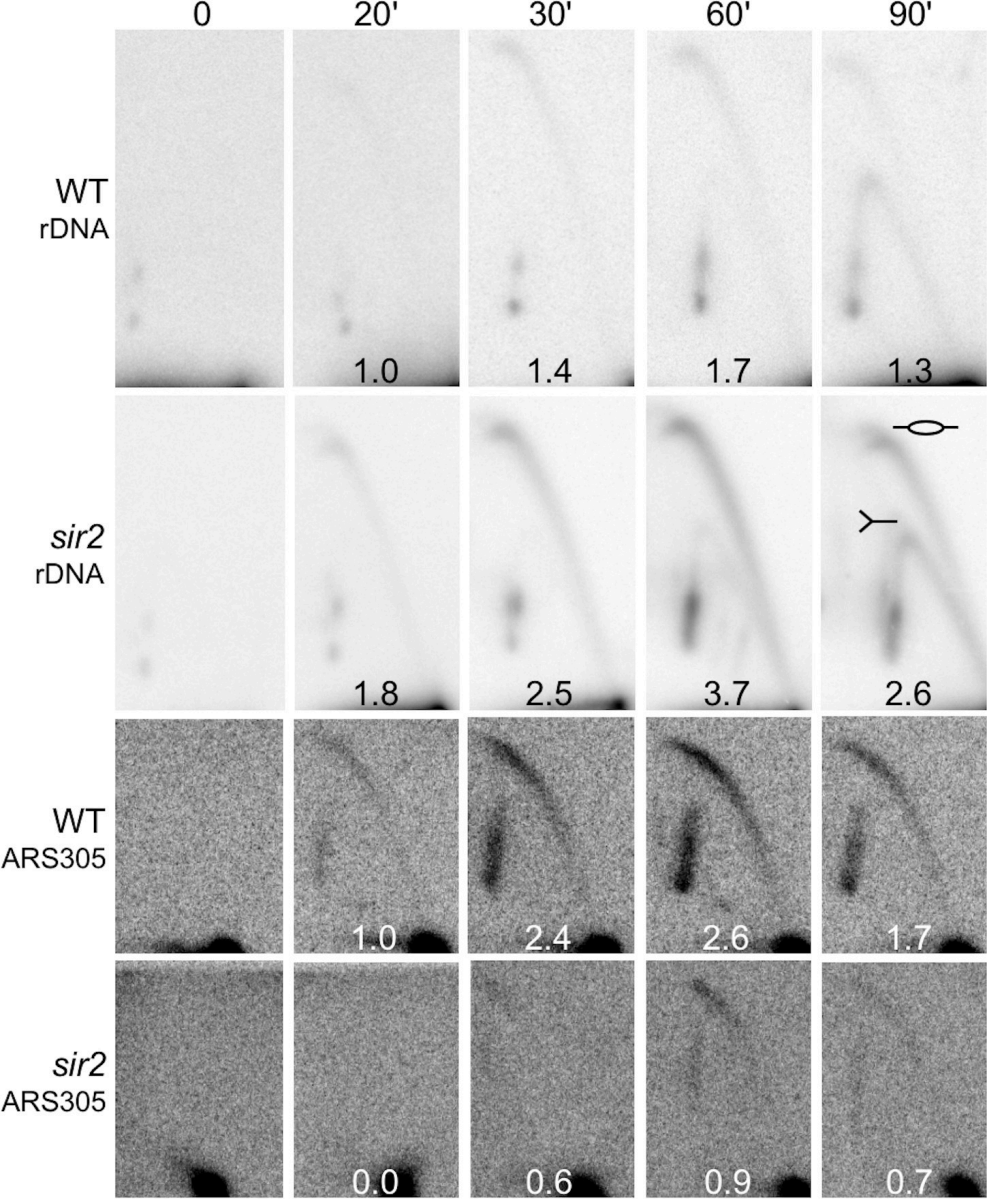
2D gel analysis of origin activity at rDNA and ARS305. WT (16535) and *sir2* (16560) cells, that carry 172 and 155 rDNA copies, respectively, were arrested in G1 with alpha factor, released into medium with 200 mM hydroxyurea, samples were collected at times 0, 20', 30', 60' and 90' and analyzed by 2D gel electrophoresis. DNA was cut with NheI for rDNA origin and EcoRV for ARS305. Replication bubble abundance was calculated by densitometry as a ratio of bubble to linear DNA signal, expressed relative to the 20’ time point in WT cells for each of the two replication origins.

To assess whether the increased rDNA replication in a *sir2* mutant still originates at approximately the same location, despite the decreased Mcm2-7 footprint, we used two-dimensional gel electrophoresis (2D gel) to compare replication intermediates at the rDNA origins in WT and *sir2* mutant cells at several time points after release from G1 arrest, with an early genomic origin (ARS305) serving as a control. 2D-gels allow assessment of the activity of specific origins because they can distinguish "bubble- shaped " replication intermediates that are formed when an origin is active from "Y-shaped" intermediates, which reflect passive replication [31]. We observed bubble signals at the rDNA in both WT and *sir2* cells, and their timing and intensity differed as expected [15, 32]: In WT cells, the bubble signal at the rDNA appeared after the control origin had fired, whereas the reverse was true in the *sir2* mutant; furthermore, maximal activation of rDNA origins was greater in *sir2* mutants compared to WT, consistent with prior reports that Sir2 suppresses activation or DNA origins (Fig 5). Excessive activation of rDNA origins in the *sir2* mutant and resulting sequestration of limiting replication factors was accompanied by reduced and delayed activation of ARS305 due to competition, consistent with previous reports [15, 32]. Although the resolution of this technique is not sufficient to distinguish initiation from Mcm2-7 complexes at their normal location at the rARS from those displaced to the right, these results demonstrate that the advanced replication at the rDNA in a *sir2* mutant initiates in the same general area as in WT.

### c-pro transcription displaces Mcm2-7 in *sir2* mutants

**Fig 6:**
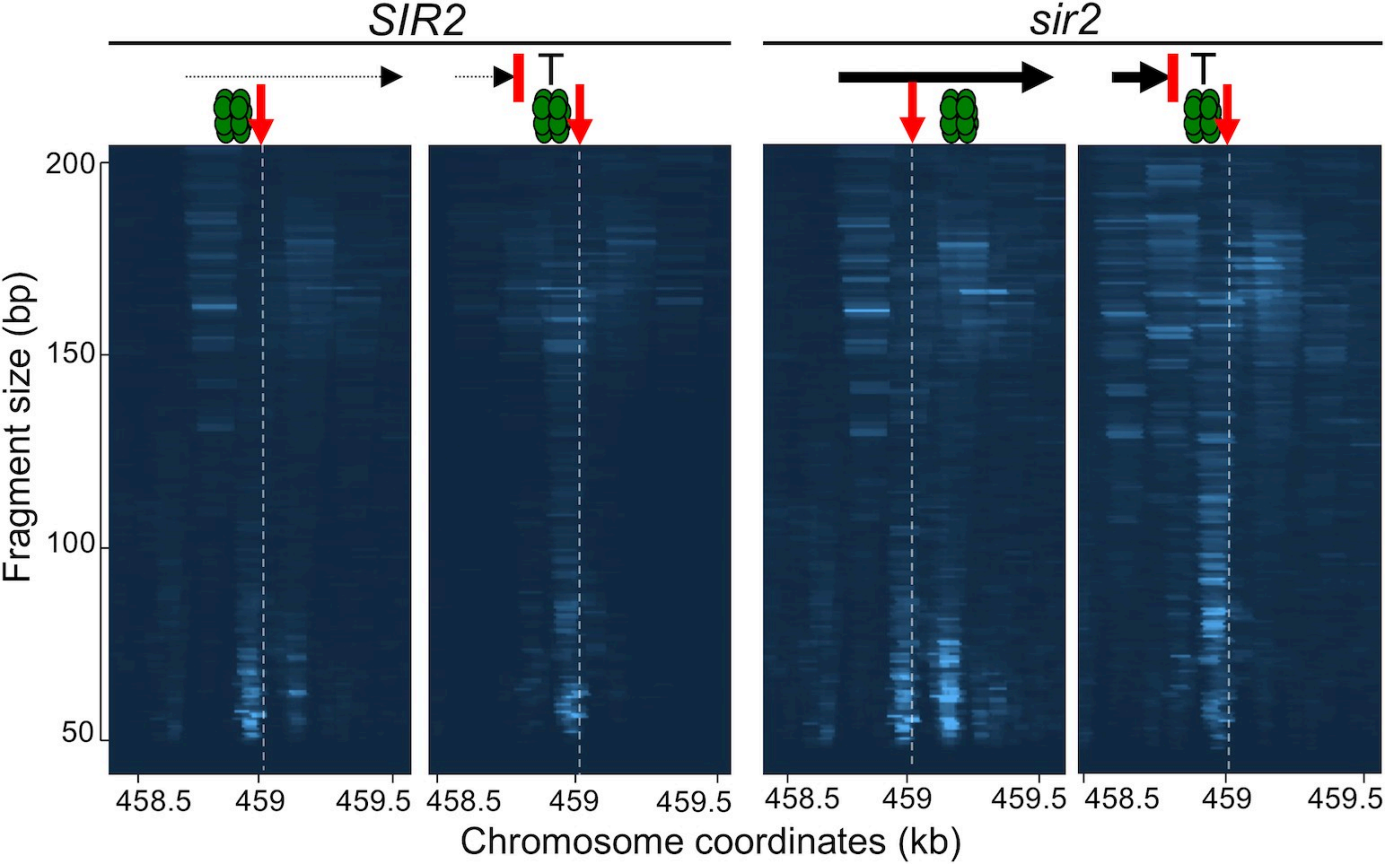
Terminator prevents displacement of Mcm2-7 (). G1-arrested *SIR2* and *sir2* strains with and without a *CYC1* transcriptional terminator (red line marked with T) (strains 16855, 16895, 16905 and 16920 from left to right with 64, 85, 170 and 81 rDNA copies, respectively), inserted between the start of c-pro transcription and the rARS, as in Fig 1A, were analyzed for Mcm2-7 distribution by Mcm2-ChEC, as in Fig 4B. qPCR of the c-pro transcript distal to the terminator demonstrated reduced transcript level (Fig 5S). Genomic coordinates according to the sacCer3 genome are on the x axis, fragment size is on the y axis, and relative read depths are indicated by color intensity, green double hexamer is Mcm2-7. The vertical dotted line and the red vertical arrow indicate the position of the rARS (base pair 458,991 in sacCer3 genome sequence).

Consistent with previous reports, we found that the level of c-pro transcript is elevated more than 20-fold in the absence of Sir2 (S3 Fig) [33–36]. Initiating approximately 200 base pairs to the left of the rARS and proceeding rightward through the origin, c-pro transcription would be expected to generate the observed rightward displacement of Mcm2-7 from their loading site (S3 Fig). If so, blocking c-pro transcription upstream of the rARS should prevent rightward displacement of Mcm2-7 complexes. To test this idea, we used a system developed by Kobayashi et al. [37] to generate a strain with transcription terminators in the rDNA array inserted between the start of c-pro transcription and the rARS, at a site marked by asterisk in Fig 1A, and we used Mcm2-MNase ChEC to monitor the location of the pre-RC (Fig 6). This system features two ribosomal repeats at the rDNA locus and the rRNA genes transcribed by RNA Pol II from a plasmid, which enables repeat alterations followed by their Fob1-mediated expansion. Our results demonstrate that premature termination of c-pro transcription (S5A Fig) with a terminator placed downstream of the c-pro transcription initiation site but upstream of the rARS prevents the rightward displacement of the pre-RC in a *sir2* mutant, thus providing strong support for our hypothesis that the passage of RNA Pol II across the replication origin, as happens in the absence of Sir2, displaces the pre-RC.

Because the terminator sequence in a *sir2* mutant prevented Mcm2-7 displacement, this system presented an opportunity to examine the effect of the terminator on rDNA replication timing. We reasoned that if displacement of Mcm2-7 is the proximal cause of early replication of the rDNA in a *sir2* mutant, and if the terminator suppresses this displacement, then the terminator should also suppress the early replication phenotype. Unfortunately, we could not test this prediction because we found that in our experimental system, the rDNA replicated early even in WT cells, regardless of the presence or absence of *SIR2* (S1 Table). The rDNA arrays in our experiment were generated by repeat expansion in a strain that contained only two repeats and no nucleolus. We suggest that the process of expansion failed to recreate an intact nucleolus, which perturbed rDNA replication timing. In support of this idea, we noted that the nucleolar staining with Nop58-GFP marker is diffuse and fragmented in the strain subjected to rDNA expansion compared with the WT strain with intact nucleolus (S5B Fig).

## Discussion

Although it has long been clear that heterochromatin is both transcriptionally silent and late replicating, the mechanism linking these two remains a mystery. On one hand, the lack of transcription could cause late replication, or *vice versa*; on the other hand, both phenomena could share a single cause [10]. Here we show that *SIR2* deletion repositions replication machinery by virtue of derepressing a Pol II-transcribed non-coding RNA adjacent to the replication origin. The Mcm2-7 is pushed ahead by advancing RNA Pol2 in the absence of *SIR2*, and this movement is blocked by a transcriptional terminator placed between the promoter and the rARS.

Pre-RCs displaced by transcription have been shown to retain their activity [18] but our results suggest that their displacement at the rDNA, as observed in *sir2* mutants, may be associated with their excessive and premature activation. How could the displacement of pre-RCs at rDNA origins increase their activation? One possibility is that displacement of pre-RCs from their initial loading site allows repeated rounds of Mcm2-7 loading, and that this in turn advances replication timing. Such a model has been proposed and is consistent with the observations that more than one Mcm2-7 complex can be loaded at a single origin, and that Mcm2-7 ChIP-seq signals are stronger at early compared to late replicating yeast origins [38]. However, quantitation of our Mcm2-7 binding data clearly demonstrate decreased, rather than increased, levels of Mcm2-7 binding at the rDNA in *sir2* mutants (Fig 3D), refuting this model. Instead, we suggest that displacement of Mcm2-7 complexes from their site of loading promotes firing by liberating them from local chromatin features that restrain origin activation. In support of this idea, at its loading site at the rARS, each Mcm2-7 complex is flanked by a well-positioned nucleosome on both sides. In contrast, most of the repositioned Mcm2-7 complexes in *sir2* cells will not have a flanking nucleosome, given the low nucleosome occupancy in that region. The absence of flanking nucleosomes could facilitate a step in origin activation subsequent to Mcm2-7 loading in *sir2* cells. It is also possible, that Mcm2-7 displacement is not the causal event, but that transcription through the origins promote their activation by another mechanism.

The majority of replication origins in budding yeast are found in intergenic regions [39, 40] between convergently transcribed genes, which is expected to reduce transcription-mediated displacement of pre-RCs. The replication origins at the *CUP1* locus, however, exhibit a striking similarity to the rDNA locus: This locus contains two or more copies of a tandem repeat, each of which contains a gene that confers copper-resistance (*CUP1*), an origin of replication, and a non-coding RNA that spans the origin (*RUF5*; RNA of Unknown Function). In contrast to the situation at the rDNA, where *SIR2* is known to repress transcription from c-pro, regulators of transcription of *RUF5* have not been identified. If such regulators are identified, it will be interesting to learn whether they advance replication timing of the *CUP1* origin and whether such organization of replication origins and non-coding RNAs constitute a recurring theme in repetitive genomic regions.

Our discovery of subtle displacement of replicative helicases by RNA Pol II in heterochromatin relied on mapping of replicative helicase at a much higher level of resolution than is attainable with more widely used approaches to chromatin analysis, such as chromatin immunoprecipitation, and provides an experimental framework for examining associations between transcription and replication during development, aging and carcinogenesis in metazoans.

## Materials and methods

### Strains and culture conditions

All yeast strains except for those with rDNA repeat expansion were in S288C background. Yeast experiments were carried out using standard YPD (yeast peptone dextrose) medium [2% (wt/vol) glucose, 1% yeast extract, 2% (wt/vol) peptone]. The full list of strains is provided in the S2 Table.

To generate *MCM2*-MNase strains, sequences containing MNase and triple FLAG tags along with KanMX selectable marker were amplified from pGZ108 tagging plasmid [29] (Addgene #70231) and inserted directly immediately upstream of the stop codon of *MCM2*.

To generate a strain with a transcriptional terminator we first introduced a marker (HygR) at the left border of the repeats in the strain TAK201 [37] by direct integration using the following primers: TAGGACATCTGCGTTATCGTTTAACAGATGTGCCGCCCCAGCCAAACTCCagattgtactgagagtgcac and AGCTTAACTACAGTTGATCGGACGGGAAACGGTGCTTTCTGGTAGATATGctgtgcggtatttcacaccg. Next, we introduced the *CYC1* terminator between the site of c-pro transcription initiation and the r*ARS*, between coordinates 458,950 and 458,951, using a "pop-in/pop-out" strategy. The strain was first transformed with the PCR product amplified from plasmid pJH105, which contains URA3 markers flanked by CYC1 terminator sequence on both sides, using the following primers: TCAGAGACCCTAAAGGGAAATCCATGCCATAACAGGAAAGTAACATCCCAgccccttttcctttgtcgatat and GAATAGTTACCGTTATTGGTAGGAGTGTGGTGGGGTGGTATAGTCCGCAT-ttacatgcgtacacgcgtttg. A resulting transformant with terminator-URA3-terminator fragment was used to generate a strain containing a single terminator by selecting URA3 pop-out events with 5-FOA. We next introduced FOB1 using a plasmid pTAK101 to enable repeat expansion. Upon the repeat expansion, we selected a strain that has lost both plasmid pTAK101 with FOB1 and plasmid pNOY353 with Pol-II transcribed 35S and 5S rDNA genes. A control strain, without terminator was also generated by repeat expansion.

### MNase-seq

MNase-seq was carried out as described [23]. Cells grown to log phase from an overnight 25 mL culture were arrested in G1 with 3 μM alpha-factor for 1.5 hrs. or G2 with 20 mg/mL nocodazole for 2.5 hrs. at 30° C. Incubations with *cdc6* temperature sensitive mutant were carried at out 23° C; log phase cells from an overnight culture were synchronized in G2 with 15 μg/mL nocodazole followed by an additional spike at 1.5 hrs. and 3 hrs. for a total 60 mg/mL. Temperature-sensitive cells were harvested after 3.5 hrs. incubation, washed twice with cold YEPD and released in 23° C or 37° C pre-warmed media with alpha-factor. Cells were arrested in G1 with 3 μM alpha-factor for 4 hrs. or 1.5 hrs. in permissive and non-permissive temperature, respectively.

Arrested cells were crosslinked with 1% formaldehyde for 30 min at room temperature water bath with shaking. Formaldehyde was quenched with 125 mM glycine and cells were centrifuged at 3000 rpm for 5 min. Cells were washed twice with water and resuspended in 1.5 mL Buffer Z (1 M sorbitol, 50 mM Tris-HCl pH 7.4) with 1 mM beta-mercaptoethanol (1.1 μL of 14.3 M beta-mercaptoethanol diluted 1:10 in Buffer Z) per 25 mL culture. Cells were treated with 100 μL 20 mg/mL zymolyase at 30° C for 20–30 min depending on cell density. Spheroplasts were centrifuged at 5000 rpm for 10 min and resuspended in 5 mL NP buffer (1 M sorbitol, 50 mM NaCl, 10 mM Tris pH 7.4, 5 mM MgCl2, 1 mM CaCl2) supplemented with 500 μM spermidine, 1 mM beta-mercaptoethanol and 0.075% NP-40. Nuclei were aliquoted in tubes with varying concentrations of micrococcal nuclease (Worthington), mixed via tube inversion, and incubated at room temperature for 20 mins. Chromatin digested with 1.9 U– 7.5 U micrococcal nuclease per 1/5th of spheroplasts from a 25 mL culture yielded appropriate mono-, di-, tri- nucleosome protected fragments for next-generation sequencing. Digestion was stopped with freshly made 5x stop buffer (5% SDS, 50 mM EDTA) and proteinase K was added (0.2 mg/ml final concentration) for an overnight incubation at 65° C to reverse crosslinking. DNA was extracted with phenol/chloroform and precipitated with ethanol. Micrococcal nuclease digestion was analyzed via gel electrophoresis prior to proceeding to library preparation. Sequencing libraries for both MNase-seq and ChEC-seq were prepared as described [21].

### ChEC-seq

Mcm2-MNase ChEC was carried out as described [29]. Briefly, cells were first permeabilized with digitonin and then treated with calcium to activate MNase; DNA was extracted with phenol/chloroform and precipitated with NaCl and ethanol.

MNase-tagged cells were grown at 30° C overnight in 50 mL cultures to 8 x 10^6^ cells/mL, arrested in G1 with 3 μM alpha-factor for 1.5 hrs, cooled on ice for 3 mins, centrifuged at 1,500 x g for 2 mins, and washed twice in cold Buffer A (15 mM Tris pH 7.5, 80 mM KCl, 0.1 mM EGTA) without additives. Washed cells were carefully resuspended in 570 μL Buffer A with additives (0.2 mM spermidine, 0.5 mM spermine, 1 mM PMSF, ½ cOmplete ULTRA protease inhibitors tablet, Roche, per 5 mL Buffer A) and permeabilized with 0.1% digitonin in 30° C water bath for 5 min. Permeabilized cells were cooled at room temperature for 1 min and 1/5th of cells were transferred in a tube with freshly made 2x stop buffer (400 mM NaCl, 20 mM EDTA, 4 mM EGTA)/1% SDS solution for undigested control. Micrococcal nuclease was activated with 5.5 μL of 200 mM CaCl2 at various times (30 sec, 1 min, 5 mins, and 10 mins) and the reaction stopped with 2x stop buffer/1% SDS. Once all time points were collected, proteinase K was added to each collected time points and incubated at 55° C water bath for 30 mins. DNA was extracted using phenol/chloroform and precipitated with ethanol. Micrococcal nuclease digestion was analyzed via gel electrophoresis prior to proceeding to library preparation. Library was prepared using total DNA, without any fragment size selection.

### Analysis of rDNA replication using S-seq

DNA replication analysis was carried out as described [13]. Briefly, cells with G1 and S-phase DNA content were isolated from logarithmically growing cells using FACS. DNA from these cells was fragmented by sonication and sequenced. rDNA replication kinetics was determined by calculating the ratio of rDNA read depths from S-phase cells and G1 cells.

### Comparison of Mcm2-7 abundance at the rDNA among strains using ChEC-seq

We calculated relative abundance of Mcm2-7 at the rDNA as the fraction of reads that map to the rDNA over the total number of reads for each strain. The relative rDNA Mcm2-7 abundances among different strains are directly compared.

### qRT-PCR analysis of c-pro transcript and rDNA copy number

RNA was extracted from logarithmically growing cells after spheroplasting using Trizol reagent and chloroform and purified after extraction with an RNA-Easy column. The sequences of the primers used in qRT-PCR for c-pro and PDA1 mRNA are provided in S3 Table. rDNA size was measured by qPCR using DNA that was extracted by phenol-choroform extraction and ethanol precipitation using the primers listed in S3 Table. As an internal standard qPCR was done in parallel on the DNA extracted from strains with 150, 90 and 30 rDNA copies whose rDNA size had been verified by Pulsed Field Gel Electrophoresis.

### Sequencing

Sequencing was performed using an Illumina HiSeq 2500 in Rapid mode employing a paired-end, 50 base read length (PE50) sequencing strategy. Image analysis and base calling was performed using Illumina's Real Time Analysis v1.18 software, followed by 'demultiplexing' of indexed reads and generation of FASTQ files, using Illumina's bcl2fastq Conversion Software v1.8.4.

### Two-dimensional plotting of the sequencing data

Sequences were aligned using the gsnap alignment software suite and data were plotted using the ggplot R package.

### 2D gel electrophoresis

2D gel electrophoresis was carried out as described [20]. Briefly, genomic DNA was prepared from 0.5% agar-embedded, G1-arrested cell or cells taken 30, 60 and 90 minutes after release into medium with 200 mmol hydroxyurea, digested with NheI (for rARS) or EcoRV (for ARS305), and subjected to 2 dimensional agarose gel electrophoresis, followed by Southern blotting.

## Supporting information

S1 Fig Reb1 footprint within *IGS2* as determined by ChEC-seq. ChEC-seq dataset for Reb1-MNase [29] was analyzed and plotted the same way as Mcm2-MNase ChEC-seq dataset in Fig 2. Genomic coordinates according to the sacCer3 genomic sequence are shown on the X-axis and the fragment size on the Y-axis.
(TIFF)

S2 Fig Insertion of the MNase tag at the C-terminus of Mcm2 does not alter growth. The strains with (16747) and without Mcm2-MNase tag (14141) were streaked out on the rich media and incubated to 48 hours at 30°.
(TIFF)

S3 Fig C-pro transcript levels in WT and *sir2*. Total RNA (not poly-A purified) was isolated from log phase cultures of WT (blue) (15691) and *sir2* (red) (15984) strains and subjected to high throughput sequencing. rDNA landmarks at the top are the same as in Fig 2.
(TIFF)

S4 Fig Mcm2-7 footprints located right of the rARS are not suppressed by *FOB1* deletion. ChEC-seq experiment was performed in biological replicas as in Fig 4B in the indicating strains. Biological replicas of each genotype demonstrate highly reproducible footprints.
(TIFF)

S5 Fig A) CYC1 terminator inserted between c-pro initiation site and rARS reduces c-pro transcript level distal to the insertion site. C-pro levels analyzed by qPCR with the primers distal to terminator insertion site were all normalized to WT. T indicates insertion of the terminator. Error bars indicate standard deviation, N = 3, ** p<0. 01 by Student’s t-test. B) A strain with expanded rDNA repeats has enlarged and fragmented nucleolus. Nop58-GFP marked nucleoli in the strain with expanded rDNA repeats (17028) are enlarged, diffuse and fragmented compared to those in the control strain (16212). White bar indicates 10 μm.
(TIFF)

S1 Table A strain with expanded rDNA repeats replicates early regardless of the *SIR2* status. rDNA replication compared to genome replication in the indicated strains presented as a fraction of reads that map to rDNA in the S phase divided with the fraction of reads that map to rDNA in the G1 phase. The S-seq analysis was carried out using datasets obtained from *SIR2* (16833) and *sir2* (16849) strains and previously published dataset of *SIR2* (15213) and *sir2* (15984) strains with WT rDNA (13).
(TIFF)

S2 Table Yeast strains. (PDF)

S3 Table Primers used for qPCR. (PDF)
