# Supplementary figures and images for: Sir2 suppresses transcription-mediated displacement of Mcm2-7 replicative helicases at the ribosomal DNA repeats
Source: PLoS Genet. 2019 May 13;15(5):e1008138. doi: 10.1371/journal.pgen.1008138 (PMC6532929; doi:10.1371/journal.pgen.1008138)

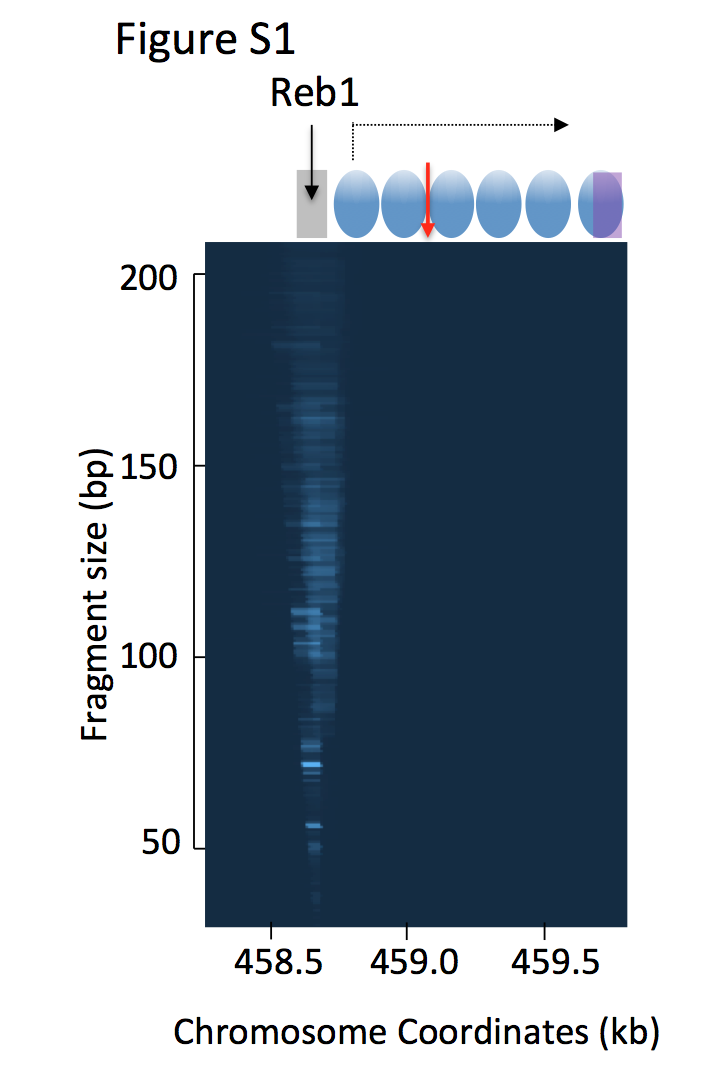

Supplement: S1 Fig — ChEC-seq dataset for Reb1-MNase [29] was analyzed and plotted the same way as Mcm2-MNase ChEC-seq dataset in Fig 2. Genomic coordinates according to the sacCer3 genomic sequence are shown on the X-axis and the fragment size on the Y-axis. (TIFF) [file pgen.1008138.s001.tiff]

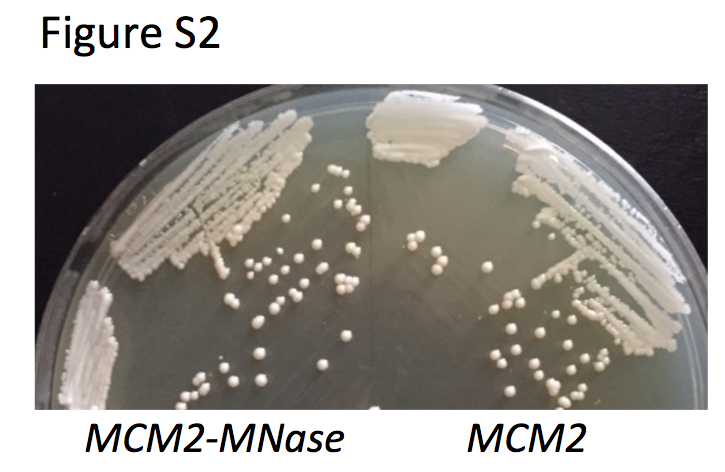

Supplement: S2 Fig — The strains with (16747) and without Mcm2-MNase tag (14141) were streaked out on the rich media and incubated to 48 hours at 30°. (TIFF) [file pgen.1008138.s002.tiff]

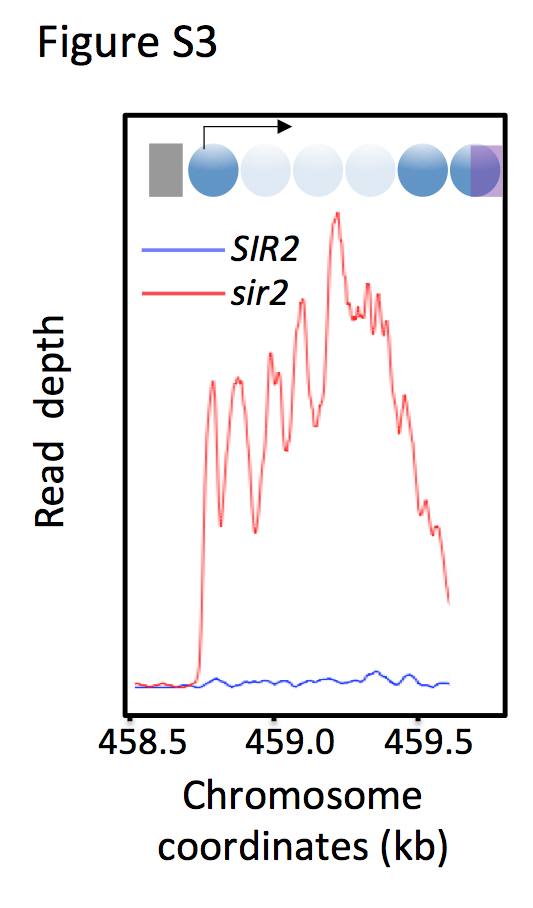

Supplement: S3 Fig — Total RNA (not poly-A purified) was isolated from log phase cultures of WT (blue) (15691) and sir2 (red) (15984) strains and subjected to high throughput sequencing. rDNA landmarks at the top are the same as in Fig 2. (TIFF) [file pgen.1008138.s003.tiff]

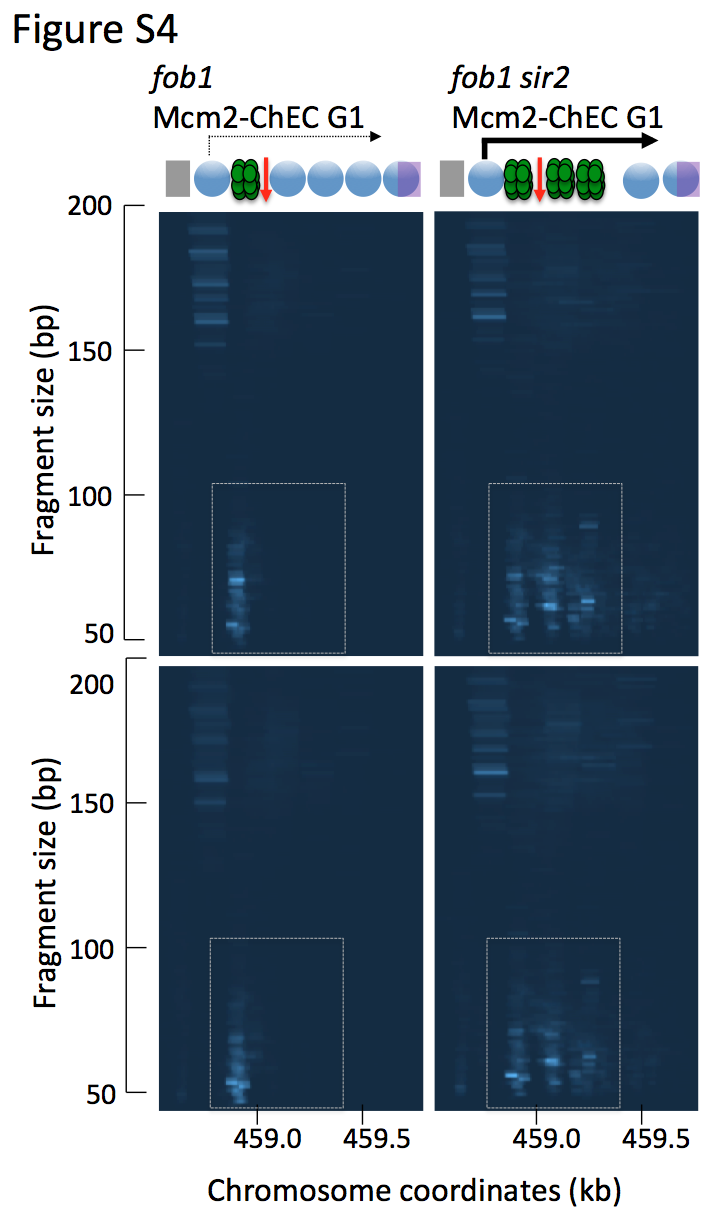

Supplement: S4 Fig — ChEC-seq experiment was performed in biological replicas as in Fig 4B in the indicating strains. Biological replicas of each genotype demonstrate highly reproducible footprints. (TIFF) [file pgen.1008138.s004.tiff]

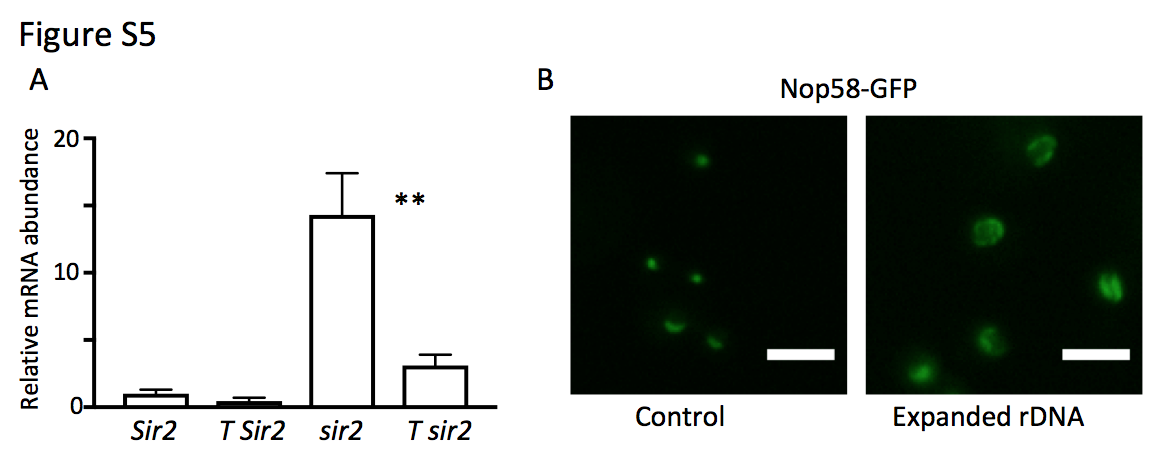

Supplement: S5 Fig — A) CYC1 terminator inserted between c-pro initiation site and rARS reduces c-pro transcript level distal to the insertion site. C-pro levels analyzed by qPCR with the primers distal to terminator insertion site were all normalized to WT. T indicates insertion of the terminator. Error bars indicate standard deviation, N = 3, ** p<0. 01 by Student’s t-test. B) A strain with expanded rDNA repeats has enlarged and fragmented nucleolus. Nop58-GFP marked nucleoli in the strain with expanded rDNA repeats (17028) are enlarged, diffuse and fragmented compared to those in the control strain (16212). White bar indicates 10 μm. (TIFF) [file pgen.1008138.s005.tiff]

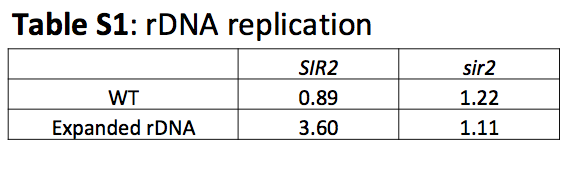

Supplement: S1 Table — rDNA replication compared to genome replication in the indicated strains presented as a fraction of reads that map to rDNA in the S phase divided with the fraction of reads that map to rDNA in the G1 phase. The S-seq analysis was carried out using datasets obtained from SIR2 (16833) and sir2 (16849) strains and previously published dataset of SIR2 (15213) and sir2 (15984) strains with WT rDNA (13). (TIFF) [file pgen.1008138.s006.tiff]
