# Supplementary material for: Sir2 suppresses transcription-mediated displacement of Mcm2-7 replicative helicases at the ribosomal DNA repeats
Source: PLoS Genet. 2019 May 13;15(5):e1008138. doi: 10.1371/journal.pgen.1008138 (PMC6532929; doi:10.1371/journal.pgen.1008138)
Supplement: S2 Table — (PDF) [file pgen.1008138.s007.pdf]

Table S2 Yeast strains

Strain	Genotype (rDNA copy number)
14141	*MATa, his3, leu2, met15, ura3* (151)
15213	*MATα, lys2Δ0, amn1Δ::NAT1* (145)
15691	*MATa, lys2Δ0, amn1Δ::NAT1*
15984	*MATα, lys2Δ0, amn1Δ::NAT1, sir2Δ::HygR* (129)
16535	*MATa, his3, leu2, met15, ura3, hmlα::HygR* (172)
16560	*MATa, his3, leu2, met15, ura3, hmlα::HygR, sir2Δ::NAT1* (155)
16212	*MATa, his3Δ1, met15Δ0, ura3Δ0, leu2Δ0, NOP58-GFP-HIS3-Mx (S. pombe his5+)*
16668	*MATa, his3, leu2, met15, ura3, sir2Δ::KanMx, hmlα::HygR* (195)
16738	*MATa, his3, leu2, met15, ura3, cdc6-1(ts)::KanMx* (180)
16747	*MATa, his3, leu2, met15, ura3, hmlα::HygR, MCM2-3xFLAG-MNase-KanMx* (133)
16769	*MATa, his3, leu2, met15, ura3, hmlα::HygR, MCM2-3xFLAG-MNase-KanMx, sir2Δ::LEU2* (162)
16818	*MATa,* chromosome XII right arm*Δ::HygR, expanded rDNA with CYC1 terminator, ade2-1, ura3-1, his3-11, trp1-1, leu 2-3,112, can1-100, fob1Δ::HIS3* (79)
16833	*MATa,* chromosome XII right arm*Δ::HygR, expanded rDNA, ade2-1, ura3-1, his3-11, trp1-1, leu 2-3,112, can1-100, fob1Δ::HIS3* (61)
16849	*MATa, ,* chromosome XII right arm*Δ::HygR, expanded rDNA, ade2-1, ura3-1, his3-11, trp1-1, leu 2-3,112, can1-100, fob1Δ::HIS3, sir2Δ::LEU2, hmlαΔ::NAT* (189)
16851	*MATa,* chromosome XII right arm*Δ::HygR, expanded rDNA with CYC1 terminator, ade2-1, ura3-1, his3-11, trp1-1, leu 2-3,112, can1-100, fob1Δ::HIS3 sir2Δ::LEU2, hmlαΔ::NAT* (72)
16855	*MATa,* chromosome XII right arm*Δ::HygR, expanded rDNA, ade2-1, ura3-1, his3-11, trp1-1, leu 2-3,112, can1-100, fob1Δ::HIS3, , MCM2-3xFLAG-MNase-KanMx* (64)
16895	*MATa,* chromosome XII right arm*Δ::HygR, expanded rDNA with CYC1 terminator, ade2-1, ura3-1, his3-11, trp1-1, leu 2-3,112, can1-100, fob1Δ::HIS3, MCM2-3xFLAG-MNase-KanMx, hmlαΔ::NAT* (85)
16905	*MATa,* chromosome XII right arm*Δ::HygR, expanded rDNA, ade2-1, ura3-1, his3-11, trp1-1, leu 2-3,112, can1-100, fob1Δ::HIS3, MCM2-3xFLAG-MNase-KanMx, sir2Δ::LEU2, hmlαΔ::NAT* (81)
16920	*MATa,* chromosome XII right arm *rDNAΔ::HygR, expanded rDNA with CYC1 terminator, ade2-1, ura3-1, his3-11, trp1-1, leu 2-3,112, can1-100, fob1Δ::HIS3, MCM2-3xFLAG-MNase-KanMx, hmlαΔ::NAT, sir2Δ::LEU2* (170)
17028	*MATa,* chromosome XII right arm*Δ::HygR, expanded rDNA, ade2-1, ura3-1, his3-11, trp1-1, leu 2-3,112, can1-100, fob1Δ::HIS3, , NOP58-GFP-KanMx* (65)
17038	*MATa, his3, leu2, met15, ura3, hmlα::HygR, MCM2-3xFLAG-MNase-KanMx, fob1Δ::NAT* (141)
17039	*MATa, his3, leu2, met15, ura3, hmlα::HygR, MCM2-3xFLAG-MNase-KanMx, fob1Δ::NAT* (165)
17040	*MATa, his3, leu2, met15, ura3, hmlα::HygR, MCM2-3xFLAG-MNase-KanMx, sir2Δ::LEU2, fob1Δ::NAT* (123)
17041	*MATa, his3, leu2, met15, ura3, hmlα::HygR, MCM2-3xFLAG-MNase-KanMx, sir2Δ::LEU2, fob1Δ::NAT* (115)

All strains except for those with expanded rDNA are in S288c background.
