# Supplementary material for: Sir2 suppresses transcription-mediated displacement of Mcm2-7 replicative helicases at the ribosomal DNA repeats
Source: PLoS Genet. 2019 May 13;15(5):e1008138. doi: 10.1371/journal.pgen.1008138 (PMC6532929; doi:10.1371/journal.pgen.1008138)
Supplement: S3 Table — (PDF) [file pgen.1008138.s008.pdf]

Table S3: Primers used for qPCR

Primer name	Sequence -5' to -3'	Purpose
5P_grDNA_2	CCGGGTAACCCAGTTCCTCA	rDNA copy number
3P_grDNA_2	TTGTATGTTCCCGCGCGTTT	rDNA copy number
5P_c_pro_qPCR	CCATCTATGTCTTCCACACC	c-PRO RNA transcript distal to termination site
3P_c_pro_qPCR	GTCATTTACAAGAGGTAGGTCG	c-PRO RNA transcript distal to termination site
PDA1_1_F	GCTGCTTCATTCAAACGCCA	Housekeeping control gene
PDA1_1_R	CTGGTGGGAGTGCGAAGAAC	Housekeeping control gene
